# Activity and rational combinations of a novel, engineered chimeric, TRAIL-based ligand in diffuse large B-cell lymphoma

**DOI:** 10.3389/fonc.2022.1048741

**Published:** 2022-10-31

**Authors:** Karolina Piechna, Aleksandra Żołyniak, Ewa Jabłońska, Monika Noyszewska-Kania, Maciej Szydłowski, Bartłomiej Żerek, Maria Kulecka, Izabela Rumieńczyk, Michał Mikula, Przemysław Juszczyński

**Affiliations:** ^1^ Department of Experimental Hematology, Institute of Hematology and Transfusion Medicine, Warsaw, Poland; ^2^ Department of Drug Discovery, Adamed Pharma S.A. Pienkow, Czosnow, Poland; ^3^ Department of Genetics, Maria Sklodowska-Curie National Institute of Oncology, Warsaw, Poland; ^4^ Department of Gastroenterology, Hepatology and Clinical Oncology, Centre for Postgraduate Medical Education, Warsaw, Poland

**Keywords:** TRAIL, apoptosis, DLBCL, venetoclax, drug resistance, endocytosis

## Abstract

**Background:**

TRAIL (TNF-related apoptosis inducing ligand) exhibits selective proapoptotic activity in multiple tumor types, while sparing normal cells. This selectivity makes TRAIL an attractive therapeutic candidate. However, despite encouraging activity in preclinical models, clinical trials with TRAIL mimetics/death receptor agonists demonstrated insufficient activity, largely due to emerging resistance to these agents. Herein, we investigated the cytotoxic activity of a novel, TRAIL-based chimeric protein AD-O51.4 combining TRAIL and VEGFA-derived peptide sequences, in hematological malignancies. We characterize key molecular mechanisms leading to resistance and propose rational pharmacological combinations sensitizing cells to AD-O51.4.

**Methods:**

Sensitivity of DLBCL, classical Hodgkin lymphoma, (cHL), Burkitt lymphoma (BL) and acute myeloid leukemia (AML) to AD-O51.4 was assessed *in vitro* with MTS assay and apoptosis tests (Annexin V/PI staining). Markers of apoptosis were assessed using immunoblotting, flow cytometry or fluorogenic caspase cleavage assays. Resistant cell lines were obtained by incubation with increasing doses of AD-O51.4. Transcriptomic analyses were performed by RNA sequencing. Sensitizing effects of selected pathway modulators (BCL2, dynamin and HDAC inhibitors) were assessed using MTS/apoptosis assays.

**Results:**

AD-O51.4 exhibited low-nanomolar cytotoxic activity in DLBCL cells, but not in other lymphoid or AML cell lines. AD-O51.4 induced death-receptor (DR) mediated, caspase-dependent apoptosis in sensitive DLBCL cells, but not in primary resistant cells. The presence of DRs and caspase 8 in cancer cells was crucial for AD-O51.4-induced apoptosis. To understand the potential mechanisms of resistance in an unbiased way, we engineered AD-O51.4-resistant cells and evaluated resistance-associated transcriptomic changes. Resistant cells exhibited changes in the expression of multiple genes and pathways associated with apoptosis, endocytosis and HDAC-dependent epigenetic reprogramming, suggesting potential therapeutic strategies of sensitization to AD-O51.4. In subsequent analyses, we demonstrated that HDAC inhibitors, BCL2 inhibitors and endocytosis/dynamin inhibitors sensitized primary resistant DLBCL cells to AD-O51.4.

**Conclusions:**

Taken together, we identified rational pharmacologic strategies sensitizing cells to AD-O51.4, including BCL2, histone deacetylase inhibitors and dynamin modulators. Since AD-O51.4 exhibits favorable pharmacokinetics and an acceptable safety profile, its further clinical development is warranted. Identification of resistance mechanisms in a clinical setting might indicate a personalized pharmacological approach to override the resistance.

## Introduction

Programmed cell death - apoptosis - is a conserved, highly controlled process, essential for the development and maintenance of homeostasis in multicellular organisms (1). Apoptosis can be triggered by extrinsic (receptor) or intrinsic (mitochondrial) pathways, both culminating in the activation of caspases, a family of enzymes cleaving a large variety of different substrates and leading eventually to cell death (2). Resistance to apoptosis allows unrestricted cell growth, and is considered a hallmark of cancer (3). Accordingly, most of the current cancer therapeutic strategies act through the induction of programmed cell death in target tumor cells. However, primary or acquired resistance to drug-induced apoptosis is a major cause of therapy failure. Thus, therapeutic reactivation/facilitation of apoptotic pathways represents a promising approach to elicit cell death in cancer cells.

TRAIL (*TNF-related apoptosis inducing ligand)*, a pro-apoptotic Tumor Necrosis Factor family member, represents an attractive strategy in this aspect owing to its several unique characteristics. Most importantly, TRAIL exhibits marked selectivity towards tumor cells, while sparing normal cells. This selectivity is related to higher expression of TRAIL receptors DR4 and DR5 (*death receptor 4 and 5*) on tumor than on normal cells, but involves multiple additional mechanisms, such as cFLIP- and XIAP-dependent inhibition of apoptosis or overexpression of TRAIL decoy receptors in normal cells (4, 5). Secondly, unlike TNF, TRAIL does not elicit shock-like symptoms after systemic administration. However, the clinical use of native TRAIL is severely limited by its short half-life (6, 7). To circumvent these limitations, a variety of TRAIL recombinant analogs or TRAIL mimetics/death receptor agonists have been developed. Their activity has been studied in clinical trials. However, these studies have demonstrated only modest clinical effects of the TRAIL-based strategies due to insufficient activity and the development of resistance (8–10).

Diffuse large B-cell lymphoma (DLBCL) is the most common type of aggressive B-cell lymphoma in adults. DLBCL exhibits highly heterogeneous clinical behavior and a complex molecular background (11–15). Depending on their transcriptomic profiles, DLBCLs can be classified into distinct categories: germinal center-like (GCB) and activated B-cell-like (ABC) subtypes, which differ also in clinical behavior (13, 16). Despite molecular heterogeneity, R-CHOP immunochemotherapy remains a standard of care in the first-line DLBCL treatment. However, this approach is ineffective in about 1/3 of patients who are either refractory to frontline therapy or relapse after the initial response, underscoring the need for better treatment modalities.

In this study, we investigated cytotoxic activity, potential resistance mechanisms and synergies of a novel, chimeric protein AD-O51.4 in DLBCL models. AD-O51.4 comprises a TRAIL-derived sequence fused to tandemly arranged VEGFA-derived peptides. The positively-charged, N-terminal VEGFA-derived peptides increase the cell surface binding of the fusion protein and thus facilitate the TRAIL portion interactions with its cognate receptors (17). Consistent with its hybrid structure, AD-O51.4 in previous studies was demonstrated to elicit dual activity: cytotoxic effects in tumor cells and antiangiogenic effects on the vascular endothelium (17). Herein, we studied AD-O51.4 activity in a broad panel of lymphoid and myeloid tumor cells lines. We show that AD-O51.4 induces apoptosis at sub-nanomolar concentrations in the sensitive DLBCL cell lines. We characterize potential targetable AD-O51.4 resistance mechanisms and propose HDAC, dynamin and BCL2 inhibitors as pharmacological modulators with a potential to restore the sensitivity to TRAIL-induced apoptosis.

## Materials and methods

### Cell culture and chemicals

Human DLBCL cell lines were maintained in RPMI-1640 (Lonza; DHL-4, DHL-6, TOLEDO, U2932, K422, RIVA, PFEIFFER) or Iscove’s Modified Dulbecco’s Medium (Lonza; Ly-1, Ly-3, Ly-4, Ly-7, Ly-18, Ly-19, HBL-1), each supplemented with 100 U/mL penicillin, 100 U/mL streptomycin (Lonza), 10% or 20% heat-inactivated fetal bovine serum (Biowest), L-glutamine (2mM, Lonza) and HEPES (10Mm, Lonza). Cell GCB- and ABC designations were determined previously (18). Cells were grown in a humidified atmosphere at 37°C with 5% CO2. AD-O51.4 was synthesized and provided by Adamed S.A. TRAIL was purchased from R&D. Dynasore, venetoclax, SAHA and panobinostat were purchased from Selleckchem. Methyl-β-cyclodextrin (MβCD) and filipin were purchased from Sigma Aldrich. Caspase 3 inhibitor (Z-DEVD-FMK), caspase 8 inhibitor (Z-IETD-FMK), caspase 9 inhibitor (Ac-LEHD-CMK) and pan-caspase inhibitor (Z-VAD-FMK) were purchased from Merck-Millipore and used at 20 μM (caspase 8 and 9 inhibitors and pan-caspase inhibitor) or 50 μM (caspase 3 inhibitor) final concentration.

### Cell viability, apoptosis and caspase activity assays

DLBCL cells were incubated on a 96-well plate with either full medium or medium with indicated inhibitors used at concentrations specified in figures and figure legends. After incubation, cell viability was assessed with the 3-(4, 5 dimethylthiazol-2-yl)-5-(3-carboxymethoxyphenyl-2-(4-sulfophenyl)-2H-tetrazolium (MTS) assay (Promega). IC_50_ values were calculated using GraphPad Prism v6.0 software. Detection of apoptosis was performed with Annexin V-FITC Apoptosis Detection Kit BD Biosciences and analyzed using FACS Canto flow cytometer (BD Biosciences). Caspase activity was measured using Caspase 3/7 Glo assay (Promega). Briefly, 0.15 ×10^6^/mL cells were incubated overnight with either AD-O51.4 or TRAIL (0.1 nM). Thereafter, Caspase Glo reagent was automatically injected to wells and the luminescence was measured using TriStar LB 941 plate reader (Berthold Technologies).

### Flow cytometry

Cells were washed with PBS and incubated with fluorochrome-conjugated mouse anti-DR4-PE, anti-DR5-PE, DcR1-PE (eBioscience) or anti-VEGFR1/2 (R&D Systems), or with control isotype-matched antibodies for 30 minutes, then washed again and analyzed using FACS Canto flow cytometer (BD Biosciences). To determine receptor changes after incubation with endocytosis modulator dynasore, cells were fixed with 4% paraformaldehyde (Polysciences) for 15 min at 37°C, chilled on ice, washed 3 times with PBS and stained with anti-DR4 and anti-DR5 antibodies as above.

### Measurement of the membrane cholesterol content

Cells were incubated for 1 hour with 10, 20 or 40 mM MβCD to elute membrane cholesterol and disrupt lipid raft integrity. Depletion of cholesterol was confirmed with filipin staining (50 ng/mL) and flow cytometry as previously described (19).

### Immunoblotting

Immunoblotting was performed as previously described (20, 21). Briefly, protein lysates were resolved by SDS-PAGE, transferred to PVDF membranes (Millipore) and immunoblotted with primary and appropriate HRP-labelled secondary antibodies (listed in **Supplemental Table 1**). Signals were developed by enhanced luminescence using ECL reagent (Perkin Elmer) and a digital image acquisition system (G:Box, Syngene). To re-probe with another antibody, blots were incubated in the stripping buffer (2%SDS, 62,5mM Tris/HCl, pH 6.8, 0,8% β-mercaptoethanol) at 50°C for 30 minutes, washed extensively in Tris-buffered saline and analyzed as described above.

### RNA sequencing

RNA was extracted using Gene MATRIX Universal RNA/miRNA Purification Kit (EURx), according to manufacturer instructions. High-quality samples (RIN≥8, determined with Bioanalyzer instrument) were enriched in poly(A)-containing mRNA using Dynabeads mRNA DIRECT Micro Kit (Thermo). The libraries were prepared with Ion Total RNA-Seq Kit v2 (Thermo) and sequenced on Ion Porton sequencer as described before (22). The raw reads were processed with Ion Torrent RNASEQ Analysis pipeline (Torrent Suite version 5.0.4) which maps reads to hg19 genome with STAR2 and bowtie2 aligners. Gene counts were generated with htseq-count version 0.6. Gene expression analysis was performed in R environment (v 4.0.4) using DESeq2, ClusterProfiler, fgsea and enrichplot packages (23, 24). Sequencing results are available *via* Gen Expression omnibus under accession number GSE208543.

### Statistical analysis

All experiments were performed in biological duplicates or triplicates as indicated in figure legends. The results show average values including standard deviations. To evaluate the differences between groups, Mann-Whitney test or Student’s *t*-test (for variables with normal distribution) were used, with p<0.05 as a significance level. Densitometric quantifications of band intensities were performed using ImageJ software (www.imagej.net) as described (25). Drug interactions were evaluated using CompuSyn software using Chou-Talalay method (26).

## Results

### Cytotoxic activity of AD-O51.4 and its mechanisms in lymphoma and leukemia cell lines

**Figure 1:**
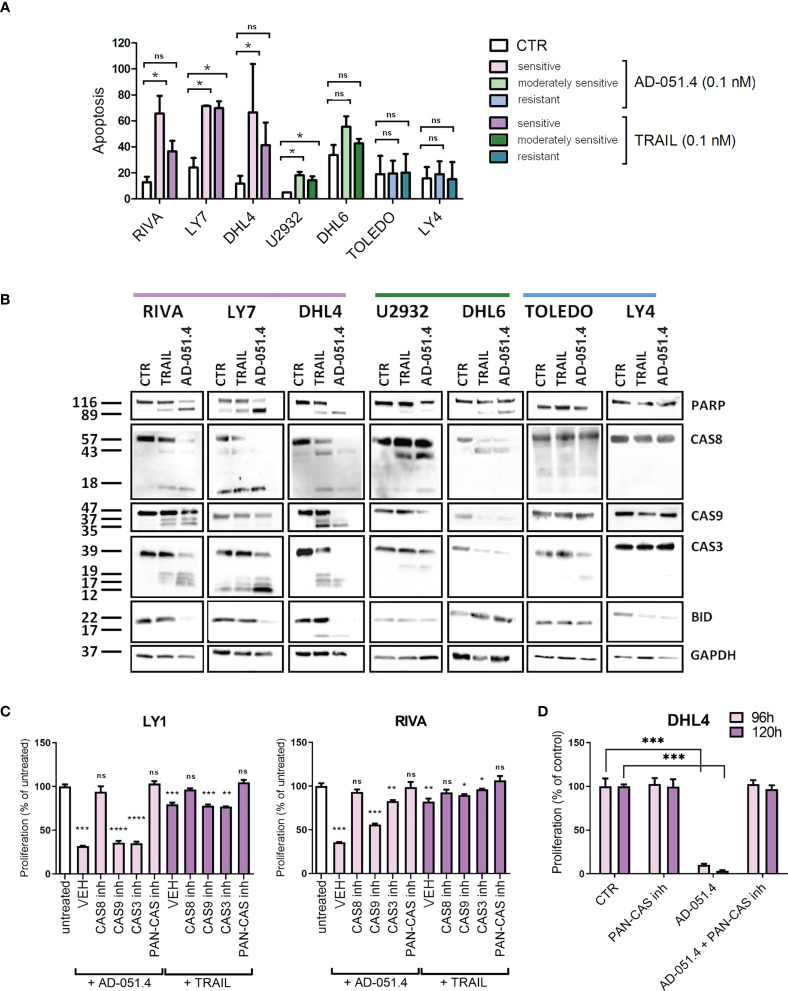
AD-O51.4 induces apoptosis in DLBCL cells. **(A)** Fraction of apoptotic cells in DLBCL cell lines incubated with 0.1 nM AD-O51.4, 0.1 nM TRAIL or PBS (control, CTR) for 24h. Early- and late–apoptotic cells are pooled together (Annexin V+/PI- and AnnexinV+/PI+, respectively). Corresponding dot-plots of a representative experiment are shown in **Supplemental Figure 3**. Experiments were performed in 2 replicates. Error bars indicate standard deviations (SD). Differences between number in apoptotic cells are indicated (Student *t*-test, *p-value<0.05, **p-value<0.005, ***p-value<0.001, ****p-value<0.0005, ns – not significant. **(B)** Processing of PARP, Caspase 8, 9, 3 and BID in DLBCL cell lines incubated with AD-O51.4 or TRAIL. Cells were incubated with the AD-O51.4 or TRAIL for 6h, lysed and processing/cleavage of indicated proteins was evaluated using immunoblotting. GAPDH served as a loading control. Cleavage of the protein is manifested either by appearance of its cleaved (lighter) form (e.g. PARP, CASP3, CASP8), or by disappearance of a full-length protein (e.g BID). **(C)** Caspase inhibition blocks induction of apoptosis in AD-O51.4-sensitive DLBCL cells. LY1 or RIVA cells were pre-incubated with caspase 3, 8 9, pan-caspase inhibitor or DMSO (vehicle, VEH) for 1 h, then with 0.1 nM AD-O51.4, 0.1 nM TRAIL for subsequent 48 h. Viability was assessed with an MTS assay. Bars represent the average of three independent experiments, error bars represent standard deviations, statistical differences are indicated as in panel A.**(D)** Prolonged incubation with AD-O51.4 does not induce caspase-independent cell death in DLBCL cells. DHL4 cells were pretreated with Z-VAD-FMK pan-caspase inhibitor (20 μM, 1 h), and then incubated with AD-O51.4 for 96-120h. Cell viability was evaluated with an MTS assay. Bars represent the average of three independent experiments, error bars represent standard deviations, statistical differences are indicated as in panel **(A)** Results were normalized to untreated cells (viability =100%).

We first evaluated the activity of the novel chimeric molecule AD-O51.4 in a panel of lymphoma and leukemia cell lines (DLBCL, Burkitt lymphoma (BL), Hodgkin lymphoma (HL) and acute myeloid leukemia (AML)). We found that AML, cHL and BL cells were resistant to AD-O51.4 and TRAIL (**Supplemental Figure 1**). In contrast, most DLBCL cell lines were sensitive to AD-O51.4 (**Figure 1A** and **Supplemental Figure 2**). Of note, TRAIL exhibited similar activity in these models. On the basis of established AD-O51.4 IC_50_ in viability assays, DLBCL cell lines were termed sensitive (IC_50_ from 0.01 nM to 0.1 nM: DHL4, LY7, RIVA), moderately sensitive (IC_50_ from 0.1 nM to 1 nM: DHL6, U2932), or resistant (IC_50_ >1 nM: LY4, TOLEDO; **Table 1** and **Supplemental Figure 2**). Twenty-four hour incubation of DLBCL cell lines with 0.1 nM AD-O51.4 markedly increased the fraction of apoptotic cells in sensitive lines, but had only moderate or no effect in moderately sensitive lines and resistant lines (**Figure 1A**, **Supplemental Figure 3**). We did not observe differences in response between GCB- and ABC-DLBCL subtypes (**Supplemental Figure 4**). To further determine the mechanism of cell death in DLBCL cells, we evaluated the expression of proteins activated in extrinsic (caspase 8), intrinsic (caspase 9, tBID), and in common apoptosis pathway (caspase 3, PARP). In sensitive lines, AD-O51.4 markedly induced caspase 3, 8 and 9 activation and tBID and PARP cleavage, indicating that extrinsic, intrinsic and effector pathways are activated in response to the drug. In contrast, resistant cell lines showed no cleavage of these proteins (**Figure 1B**). Since death receptors can trigger cell death in caspase-independent mechanisms [e.g *via* necroptosis (27)], we next assessed whether AD-O51.4 cytotoxicity requires caspase activation. In these experiments, caspase 8 or pan-caspase inhibition blocked cell death induced by AD-O51.4 entirely, even with extended incubation times (up to 120h; **Figures 1C, D**). These studies demonstrate that the extrinsic apoptotic pathway is the principal cell death mechanism triggered by AD-O51.4.

**Table 1:** AD-O51.4 and TRAIL half maximal inhibitory concentrations (IC_50_) in DLBCL cell lines.

DLBCL cell line	AD-O51.4 IC_50_ [nM]	TRAIL IC_50_ [nM]
**DHL4**	0.012	0.014
**K422**	0.011	0.066
**LY1**	0.047	0.04
**LY7**	0.04	0.039
**RIVA**	0.05	0.013
**LY18**	0.042	0.17
**LY19**	0.028	0.564
**HBL1**	0.07	0.13
**DHL6**	0.14	0.17
**U2932**	0.25	>1
**LY4**	>1	>1
**TOLEDO**	>1	>1
**PFEIFFER**	>1	>1
**LY3**	>1	>1

Cells with IC_50_ less than 0.1 nM were considered highly sensitive, cells with IC_50_ between 0.1 and 1 nM were considered moderately sensitive, and cells with IC_50_ greater than 0.1 nM were considered resistant.

**Figure 2:**
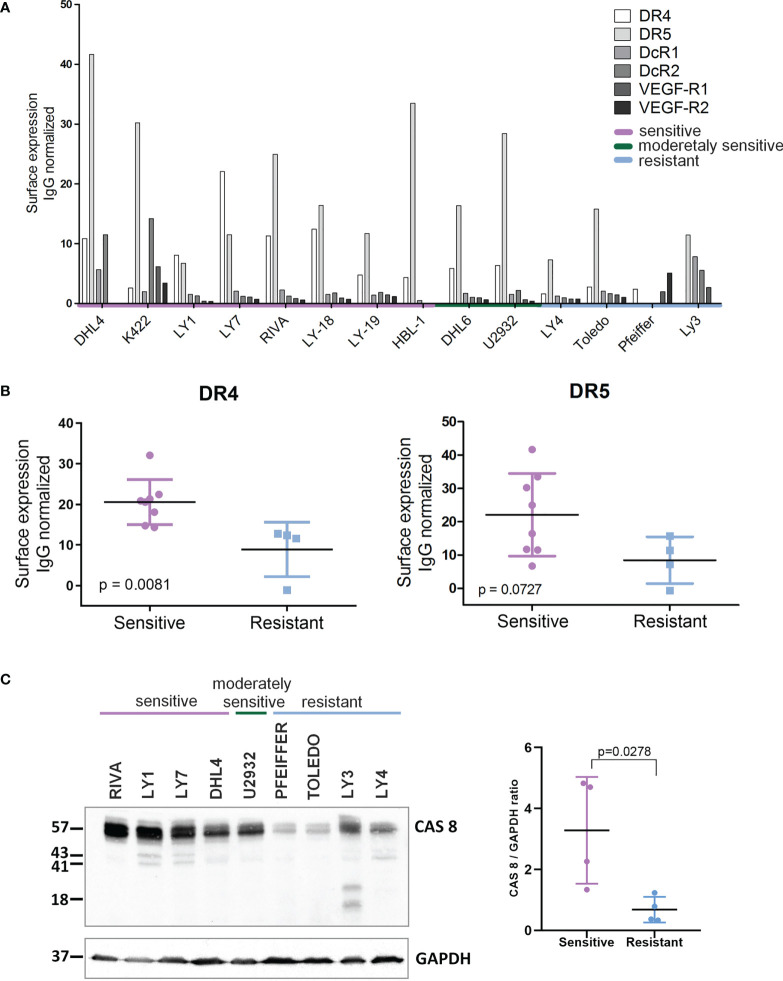
Expression of death receptors and caspase 8 is higher in AD-O51.4 –sensitive DLBCL cell lines. **(A)** Expression of death receptors 4 and 5 (DR4, DR5) decoy receptors 1 and 2 (DcR1, DcR2) and VEGF receptors 1 and 2 (VEGF-R1, VEGF-R2) was assessed in DLBCL cell lines using FACS and appropriate fluorochrome – conjugated antibodies. Bars indicate isotype control-normalized mean fluorescence values for each receptor. The bar plot represents the representative of three independent experiments. **(B)** Comparison of isotype-control normalized MFI values of DR4 and DR5 for AD-O51.4 -sensitive and –resistant DLBCL cell lines. **(C)** Left panel: expression of caspase 8 in AD-O51.4 -sensitive and –resistant DLBCL cell lines was assessed by immunoblotting. GAPDH served as a loading control. Right panel: CASP8 band intensities were quantified using pixel densitometry and normalized to GAPDH levels. Differences between sensitive and resistant cell lines were calculated using Student *t*-test.

These findings prompted us to determine whether the sensitivity of DLBCL cells to AD-O51.4 depends on the DR4/DR5 or caspase 8 expression. Sensitive cell lines showed significantly higher surface expression of DR4 (p=0.081) and markedly higher expression of caspase 8 (p=0.028, **Figure 2**). Of note, other TRAIL surface receptors (decoy receptors 1 and 2) were expressed at very low levels. Since AD-O51.4 includes VEFG-derived peptide domains, we also determined the VEGFR1 and VEGFR2 expression on the surface of DLBCL cell lines. Expression of these receptors was low or undetectable, indicating that their role in the AD-O51.4 cytotoxicity in DLBCL cells is unlikely.

Previous reports demonstrated that in certain B-cell malignancies, the membrane microarchitecture and constitutive localization of death receptors in lipid rafts are required for TRAIL-induced apoptosis (28). To determine whether the same spatial arrangement is required for AD-O51.4 activity in DLBCL cell lines, we used a cholesterol-eluting and lipid rafts disrupting agent, methyl-β-cyclodextrin (MβCD). In sensitive DLBCL cells LY1 and LY7, MβCD effectively depleted cholesterol from the cell membrane, but did not affect AD-O51.4 activity (**Supplemental Figure 5**).

### Mechanisms of acquired AD-O51.4 resistance

**Figure 3:**
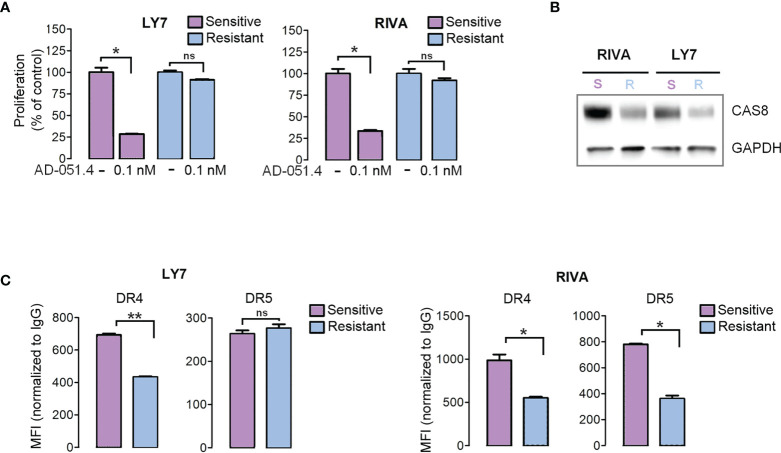
Engineered AD-O51.4 - resistant DLBCL cell lines have decreased caspase 8 and death receptor expression. **(A)** LY7 and RIVA cell lines were incubated with increasing doses of AD-O51.4 until reached complete resistance to 0.1 nM AD-O51.4. The viability of parental (purple bars) and engineered resistant (blue bars) cells is shown. Bars indicate the average of three experiments and error bars represent standard deviations. Results were normalized to untreated (control) cells. **(B)** Expression of caspase 8 in parental *vs* resistant LY7 and RIVA cells was assessed by immunoblotting. **(C)** Surface expression of DR4 and DR5 in parental *vs* resistant LY7 and RIVA cells was assessed by flow cytometry. Boxes represent average MFI from 3 replicates normalized to isotype-matched antibody, error bars represent standard deviations. *p-value<0.05, **p-value<0.005, ns – not significant.

**Figure 4:**
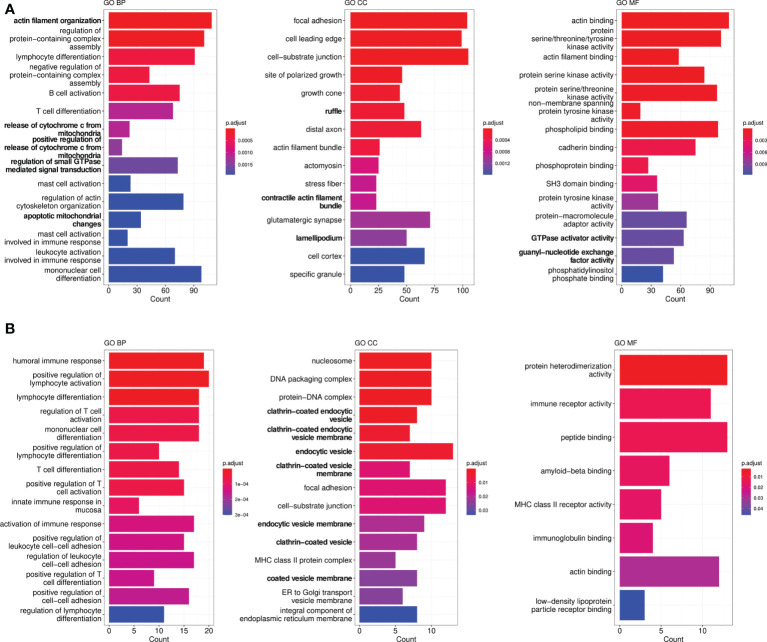
Gene ontology (GO) enrichment analysis in genes differentiating sensitive (parental) and engineered resistant LY7 **(A)** and RIVA cells **(B)**. Graphs demonstrate top significantly enriched GO CC (*cellular component*), GO BP (*biological process*) and GO MF (*molecular function*) terms.

**Figure 5:**
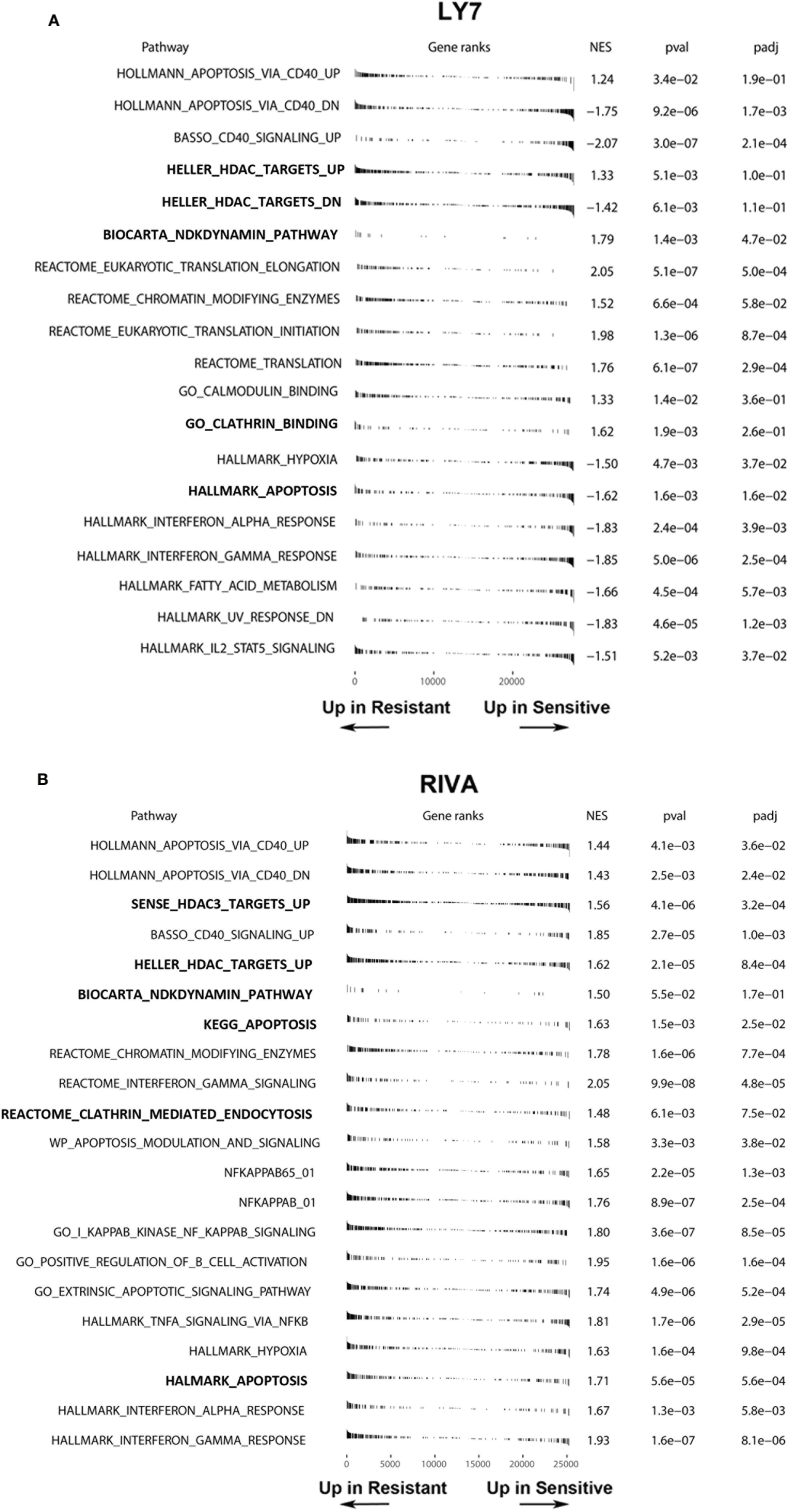
Gene set enrichment analysis showing differentially expressed gene sets in parental (sensitive) and engineered resistant LY7 **(A)** and RIVA **(B)** cells. Normalized enrichment scores, raw and adjusted p-values of apoptosis, endocytosis and HDAC-related gene-sets are shown. Plots indicate the positions of genes from a given gene set in a list of differentially expressed genes ranked by the value of Wald statistics. NES, Normalized Enrichment Score; padj, adjusted p-value.

To define the molecular mechanisms associated with AD-O51.4 resistance, we first developed drug-resistant isogenic cell lines by incubating sensitive RIVA and LY7 lines with increasing concentrations of AD-O51.4 until they reached complete resistance to 0.1 nM of AD-O51.4 (**Figure 3A**). Similar to cells with primary resistance (**Figure 2**), cells with acquired resistance exhibited decreased caspase 8 and DR4 expression (**Figures 3B, C**). DR5 expression decreased in RIVA, but not in resistant LY7 cells (**Figure 3C**). Thereafter, to understand the mechanisms of resistance in DLBCL cell lines in an unbiased manner and without *a priori* hypotheses, we compared gene expression profiles of parental and resistant cell lines using RNA sequencing and analyzed the gene ontology term enrichment in the genes differentially expressed (adjusted p value <0.05) between isogenic sensitive and resistant lines (**Figure 4**). In these analyses, we noted the enrichment of genes associated with cell membrane dynamics (membrane ruffle and lamellipodia formation, clathrin-mediated endocytosis, endocytic vesicle transport, cytoskeleton reorganization, protein membrane trafficking, GTP-ase activity), and regulation of apoptosis. To further understand the mechanisms of acquired resistance to AD-O51.4, we performed Gene Set Enrichment Analysis (GSEA; **Figure 5**). Consistent with the results of GO term enrichment, these analyses confirmed that AD-O51.4-resistant cells are characterized by overexpression of gene sets associated with clathrin-mediated endocytosis, dynamin pathway and cytoskeleton reorganization, suggesting that AD-O51.4 resistance might be acquired through increased receptor endocytosis. Resistant cells also showed differential expression of genes associated with apoptosis, indicating that modulation of apoptosis executing proteins might be another mechanism leading to resistance. Third, resistant cells demonstrated differential expression of HDAC-dependent genes. Since epigenetic mechanisms facilitate adaptive reprogramming of gene expression in response to various stress stimuli, including cytotoxic drugs, and are responsible for cell phenotypic plasticity (29), we hypothesized that epigenetic changes might be also involved in acquisition of AD-O51.4 resistance. Importantly, since increased endocytosis of death receptors, modulation of the apoptosis-controlling genes and/or epigenetic reprogramming can be pharmacologically targeted, we hypothesized that modulation of these pathways would restore the AD-O51.4 sensitivity.

### Pharmacological modulation of AD-O51.4 resistance in DLBCL models

**Figure 6:**
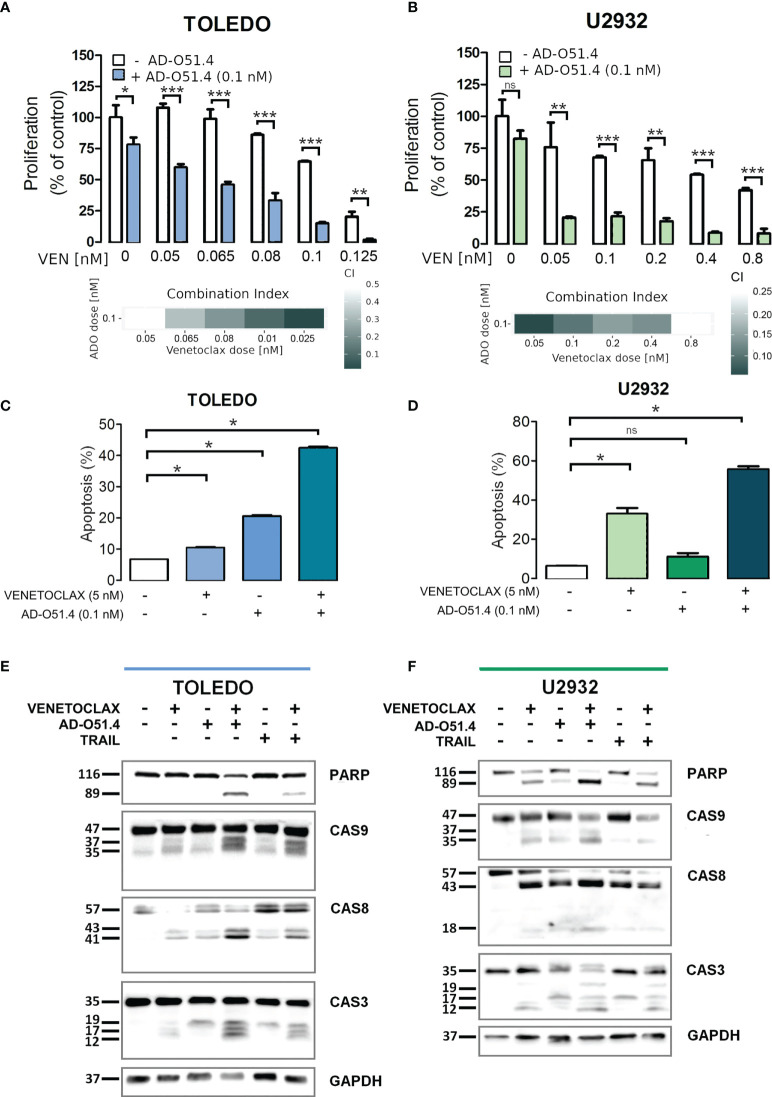
Venetoclax sensitizes resistant DLBCL cells to AD-O51.4 **A, B**. Primary resistant TOLEDO and U2932 cells were preincubated (5h) with indicated doses of venetoclax and then treated with AD-O51.4 for 72 h. Cell viability was assessed using an MTS assay in triplicates. Results were normalized to control (untreated) cells. Combination indexes (CI) for all dose combinations are indicated below the plots. **C, D.** Increased apoptosis in primary resistant TOLEDO and U2932 cells incubated with a combination of venetoclax and AD-O51.4. Cells were preincubated (5h) with 5 nM venetoclax and subsequently treated with z 0.1 nM AD-O51.4 for 24 h. Apoptosis was assessed using Annexin V/PI staining. Bars indicate the average of the combined fraction of early and late apoptotic cells (PI-/AnnexinV+ and PI+/AnnexinV+, respectively) from three replicates. Error bars indicate standard deviations. **E, F.** Expression and cleavage of caspase 3, 8, 9 and PARP in TOLEDO and U2932 cells after 1 h of preincubation with venetoclax (5 nM) and subsequent 5 h treatment with 0.1 nM AD-O51.4. In panels A-D, statistical differences were evaluated by *t*-test; *p-value<0.05, **p-value<0.005, ***p-value<0.001, ns – not significant.

**Figure 7:**
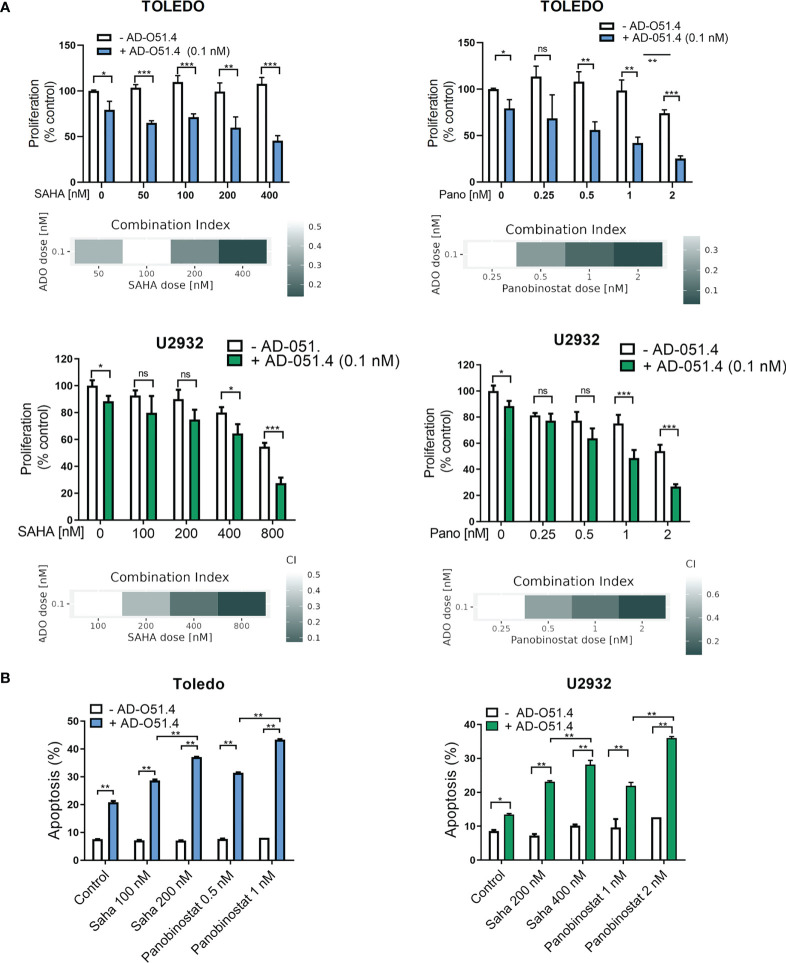
HDAC inhibitors sensitize resistant DLBCL cells to AD-O51.4. **(A)** Primary resistant TOLEDO and U2932 were preincubated with HDAC inhibitors SAHA (100 to 800 nM) or panobinostat (0.25 to 2 nM) or DMSO (control) for 24 h. Thereafter, cells were treated with 0.1 nM AD-O51.4 or PBS for additional 48 h. Cell viability was assessed using an MTS assay in triplicates. Bars and error bars indicate averages and standard deviations, respectively. Results were normalized to untreated cells (control). Combination indexes (CI) for all dose combinations are indicated below the plots. **(B)** Cells were treated as in A for 24h, and apoptosis was determined using Annexin V/PI staining. Bars indicate the average of the combined fraction of early and late apoptotic cells (PI-/AnnexinV+ and PI+/AnnexinV+, respectively) from two replicates. Error bars indicate standard deviations. In panels A-B, statistical differences were evaluated by *t*-test; *p-value<0.05, **p-value<0.005, ***p-value<0.001, ns – not significant.

To verify these hypotheses, we first tested the synergy between AD-O51.4 and a proapoptotic BCL2 inhibitor, venetoclax. In these experiments, we used a resistant cell line TOLEDO and a moderately sensitive line U2932. As predicted, venetoclax synergized with AD-O51.4 (Combination Index [CI]<0.5, for all dose combinations, **Figures 6A-D**). To confirm these observations, we assessed the biochemical markers of AD-O51.4-induced apoptosis in these cells. While venetoclax or AD-O51.4 induced weak or no PARP and caspase cleavage, the combination of these drugs induced a markedly increased abundance of cleaved forms of these markers (**Figures 6E-F**). Next, we asked whether HDAC inhibitors would sensitize resistant cells to AD-O51.4-mediated apoptosis. For these experiments, we used pan-HDAC inhibitors - SAHA and panobinostat. While each of the HDAC inhibitors showed little activity when used as a single agent, they markedly sensitized resistant/moderately sensitive DLBCL cell lines to AD-O51.4 (CI<0.54 and CI<0.52 for SAHA + AD-O51.4 combinations in Toledo and U2932, respectively; CI<0.44 and CI<0.77 for panobinostat + AD-O51.4 combinations in Toledo and U2932, respectively; **Figure 7**).

**Figure 8:**
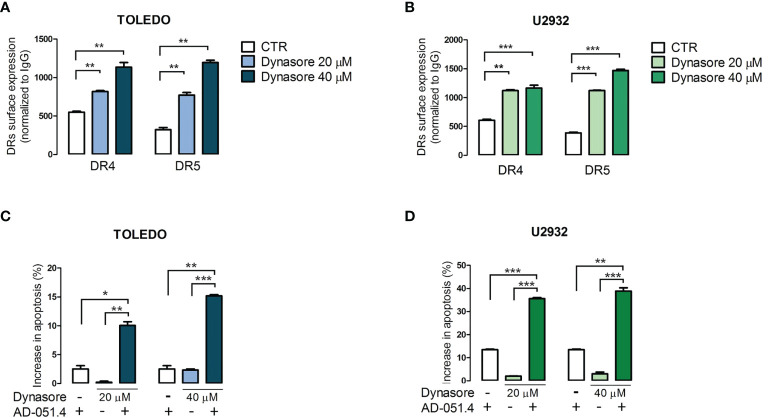
Dynamin inhibition increases the surface expression of death receptors and increases sensitivity to AD-O51.4. **A, B.** Dynamin inhibitor dynasore increases surface DR4 and DR5 expression in TOLEDO and U2932 in a dose-dependent manner. Cells were incubated with DMSO (control) or 20 - 40 μM dynasore for 30 minutes and fixed to prevent further endocytosis/recycling of DR4 and DR5. The expression of death receptors was assessed by flow cytometry. Bars indicate mean fluorescence intensity (MFI) normalized to isotype-matched antibody. Data represents an average of 3 experiments, error bars represent standard deviations. **C, D.** Inhibition of endocytosis sensitizes primary resistant TOLEDO and U2932 cells to AD-O51.4 -induced apoptosis. Cells were pretreated with dynasore (20 - 40 μM) for 1 h and treated with 0.1 nM AD-O51.4 for 24 h. Apoptosis was determined using Annexin V/PI staining. Bars indicate the average of the combined fraction of early and late apoptotic cells (PI-/AnnexinV+ and PI+/AnnexinV+, respectively) from two replicates. Error bars indicate standard deviations. In panels A-D, statistical differences were evaluated by *t*-test; *p-value<0.05, **p-value<0.005, ***p-value<0.001, ns – non significant.

Finally, we determined whether modulation of clathrin-mediated endocytosis increases AD-O51.4 activity. To test this hypothesis, we used dynasore, a cell-permeable, non-competitive inhibitor of GTPase activity of dynamin 1 and 2 (DNM1/2), essential for clathrin- coated vesicle formation and subsequent scission of nascent endosome (30). As expected, dynasore increased surface expression of DR4 and DR5 in resistant/moderately sensitive cell lines TOLEDO and U2932 in a dose-dependent manner (**Figures 8A, B**). Importantly, dynasore used as a single agent in these experiments showed no cytotoxic activity over short incubation periods. Consistent with the increased death receptor expression, dynasore-pretreated TOLEDO and U2932 cells exhibited dramatically increased levels of apoptosis (**Figures 8C, D**).

## Discussion

TRAIL exhibits unique proapoptotic activity against a variety of tumor cells while sparing non-transformed cells. These characteristics placed the TRAIL ligand-receptor system in the spotlight as a potential cancer therapy and multiple TRAIL mimetics were evaluated in clinical trials. Results of these studies generally demonstrated acceptable toxicity, but the limited activity of TRAIL-based approaches due to the rapid development of resistance. The resistance to TRAIL mimetics/analogs can emerge in several mechanisms. First, since TRAIL requires death receptors 4 and 5 (DR4/5) for activity, loss of the surface receptors, their post-translational modifications, changes in lipid rafts or induction of decoy receptors expression confers resistance to TRAIL-based therapies. Second, blockades in TRAIL-initiated apoptotic signal transduction or induction of anti-apoptotic proteins attenuate TRAIL therapeutic activity. Third, multiple transcriptional and epigenetic mechanisms, triggered in response to TRAIL signaling, can program tumor cells for TRAIL or TRAIL mimetics resistance.

Since TRAIL/TRAIL mimetics exhibit a very attractive safety profile, despite the limited efficacy and development of resistance, TRAIL-based therapeutic strategies remain still in the focus of researchers and clinicians as a potential therapeutic strategy. The reversible nature of at least some of TRAIL resistance mechanisms leaves a relatively broad space for sensitization to TRAIL or TRAIL mimetics. Detailed characterization of these mechanisms and identification of potential targetable vulnerabilities in preclinical models is crucial for future precise pharmacological interventions restoring sensitivity.

In this study, we evaluated a newly developed TRAIL mimetic, AD-O51.4. AD-O51.4 is a hybrid protein, composed of TRAIL-derived DR ligand fused to N-terminal VEGF derived, positively charged peptide. This molecule exhibits several unique characteristics making it a promising clinical candidate. First, AD-O51.4 exhibits favorable pharmacokinetics, including extended plasma half-life, large volume of distribution and preferential accumulation in tumors (17). In preclinical models, AD-O51.4 demonstrated a very good safety profile – neither mice nor monkeys treated with AD-O51.4 demonstrated symptoms of drug toxicity (17). AD-O51.4 exhibited promising toxicity in solid tumor models (cell lines and patient derived xenografts - lung adenocarcinoma, colorectal, large-cell lung, esophageal, pancreatic, bladder, and kidney cancers, hepatoblastoma and osteosarcoma) (17). Recently, the efficacy of AD-O51.4 has been also demonstrated in a broad panel of colorectal cancer cell lines and patient derived xenografts (31). These studies also highlighted the unique mechanism of action of AD-O51.4, combining death receptor signaling with DR-unrelated mechanism, driven by VEGF-derived portion of AD-O51.4, which induced FAK (focal adhesion kinase) signaling and remodeling of the actin cytoskeleton (17). The VEGF-derived AD-O51.4 portion also suppressed angiogenesis *in vitro* and *in vivo* (17).

Herein, we demonstrate that AD-O51.4 exhibits high activity in several DLBCL cell lines. We demonstrated that DR4/5 expression was higher in sensitive cells and the caspase activation is crucial for AD-O51.4 activity, while caspase-independent cell death pathways are not involved. In solid tumors, binding of the VEGF-derived, positively charged N-terminal portion of AD-O51.4 to the cell surface led to FAK activation and abnormalities in the actin cytoskeleton, characteristic of integrin mediated death. Similarly to DR-induced apoptosis, integrin-mediated death involves activation of caspase 8, although in complexes with actin and integrins, not with FADD and DRs (32). Consistent with this, neutralization of the positive charge of AD-O51.4 attenuated its proapoptotic activity in solid tumor models (17). However, given the low expression of VEGFR in DLBCL cells, VEGFR-mediated activity in these tumors is unlikely. Regardless of the upstream mechanism triggered by AD-O51.4 in DLBCL, apoptosis initiated by this ligand requires high expression of caspase 8. This is consistent with previous studies, which demonstrated resistance of cell lines with low caspase 8 expression to TRAIL (33, 34).

Consistent with previous clinical experience with TRAIL analogues, several DLBLC cell line models exhibited primary resistance to AD-O51.4. We also demonstrated that the resistance can be acquired through repeated/extended exposure to the drug, mimicking acquired resistance during therapy. To identify potential strategies sensitizing to AD-O51.4, we characterized the key biological mechanisms driving primary and acquired resistance in these cells. We demonstrated that AD-O51.4 sensitivity can be increased by blocking DR internalization (dynasore), blocking BCL2 antiapoptotic activity (venetoclax) or by modulating epigenetic mechanisms, such as histone acetylation (SAHA, panobinostat). Although endocytosis inhibitors used in these studies are “tool” compounds, unlikely to enter clinical trials, there are clinically available modulators of clathrin-dependent endocytosis. For example, certain phenothiazine derivatives, such as chloropromazine, inhibit dynamin 1 and 2 GTP-ase activity at clinically achievable concentrations. Given the critical role of dynamins in the scission of nascent vesicles and endocytosis, such an approach appears to be a rational strategy to increase DR density on target cells.

Since the identified resistance mechanisms are likely operating redundantly in the same cells, and are likely susceptible to clonal selection, triple- and higher-order drug combinations might exhibit synergistic AD-O51.4 (re)sensitizing effect. Importantly, numerous studies of combinations of TRAIL analogues with empirical chemotherapeutics/targeted agents indicated that these strategies are generally well tolerated. However, these studies were not driven by biomarkers and involved all-comers. Given the empirical nature of these combinations, ignorant to individual and sometimes redundant molecular mechanisms driving resistance, it is not surprising that they also exhibited limited activity. Thus, future strategies involving TRAIL analogues including AD-O51.4, should be more personalized, biomarker and knowledge-driven. For example, based on the preclinical data presented herein - low caspase 8 expression might represent the biomarker of resistance and exclusion criterion. Second, given the mechanism of action and cellular plasticity leading to resistance, the therapeutic strategies should be designed upfront to maximize the response and eliminate tumor cells before resistance occurs. This goal can be achieved by rational combinations, such as presented herein. Individualized therapeutic approaches based on such principles are most likely to increase the success rate of TRAIL-based clinical trials.

## Data availability statement

The datasets presented in this study can be found in online repositories. The names of the repository/repositories and accession number(s) can be found below: https://www.ncbi.nlm.nih.gov/geo, accession number GSE208543.

## Author contributions

KP designed experiments, performed research and wrote the manuscript. AŻ interpreted results and wrote the manuscript. EJ designed experiments and performed research. MN-K designed experiments and performed research. MS designed experiments, performed research and interpreted data. BŻ designed experiments, acquired funding and interpreted data. MK analyzed data. IR performed RNA-Seq. MM analyzed data. PJ acquired funding, designed experiments, performed research, analyzed data, wrote the manuscript. All authors contributed to the article and approved the submitted version.

## Funding

The study was funded by the National Center for Research and Development grant STRATEGMED2/265566/6/NCBR/2015.

## Acknowledgments

The authors would like to thank ADAMED S.A. for providing AD-O51.4.

## Conflict of interest

BŻ is an ADAMED S.A. employee.

The remaining authors declare that the research was conducted in the absence of any commercial or financial relationships that could be construed as a potential conflict of interest.

## Publisher's note

All claims expressed in this article are solely those of the authors and do not necessarily represent those of their affiliated organizations, or those of the publisher, the editors and the reviewers. Any product that may be evaluated in this article, or claim that may be made by its manufacturer, is not guaranteed or endorsed by the publisher.
