# Supplementary material for: Activity and rational combinations of a novel, engineered chimeric, TRAIL-based ligand in diffuse large B-cell lymphoma
Source: Front Oncol. 2022 Oct 31;12:1048741. doi: 10.3389/fonc.2022.1048741 (PMC9659889; doi:10.3389/fonc.2022.1048741)

Supplementary Material

**Activity and rational combinations of a novel, engineered chimeric, TRAIL-based ligand in diffuse large B-cell lymphoma**

**Karolina Piechna^1^, Aleksandra Żołyniak^1^, Ewa Jabłońska^1^, Monika Noyszewska-Kania^1^, Maciej Szydłowski^1^, Bartłomiej Żerek^2^, Maria Kuleck­a^3,4^, Izabela Rumieńczyk^3^, Michał Mikula^3^, Przemysław Juszczyński^1^**

**Supplemental Table 1.** Antibodies used in the study

| Name | Manufacturer | Dilution | Catalog # |
| --- | --- | --- | --- |
| PARP | Cell signaling | 1:1000 | 9542 |
| BID | Cell signaling | 1:1000 | 2002 |
| Caspase-3 | Cell signaling | 1:1000 | 9662 |
| Caspase-8 | Cell signaling | 1:1000 | 4790 |
| Caspase-9 | Cell signaling | 1:1000 | 9502 |
| BCL2 | BD Biosciences | 1:1000 | 610539 |
| XIAP | Cell Signaling | 1:1000 | 2042 |
| BCL-X_L_ | Cell Signaling | 1:1000 | 2762 |
| GAPDH | Sigma Aldrich | 1:2000 | CB1001 |
| MCL1 | Cell Signaling | 1:1000 | 5453 |
| Anti-rabbit IgG -Peroxidase | Sigma Aldrich | 1:5000 | A0545 |
| Anti-mouse IgG -Peroxidase | Sigma Aldrich | 1:10000 | A9044 |

**Supplemental Figure 1**. AD-O51.4 and TRAIL dose-response curves and IC50 values in Hodgkin lymphoma, Burkitt lymphoma and AML cell lines.


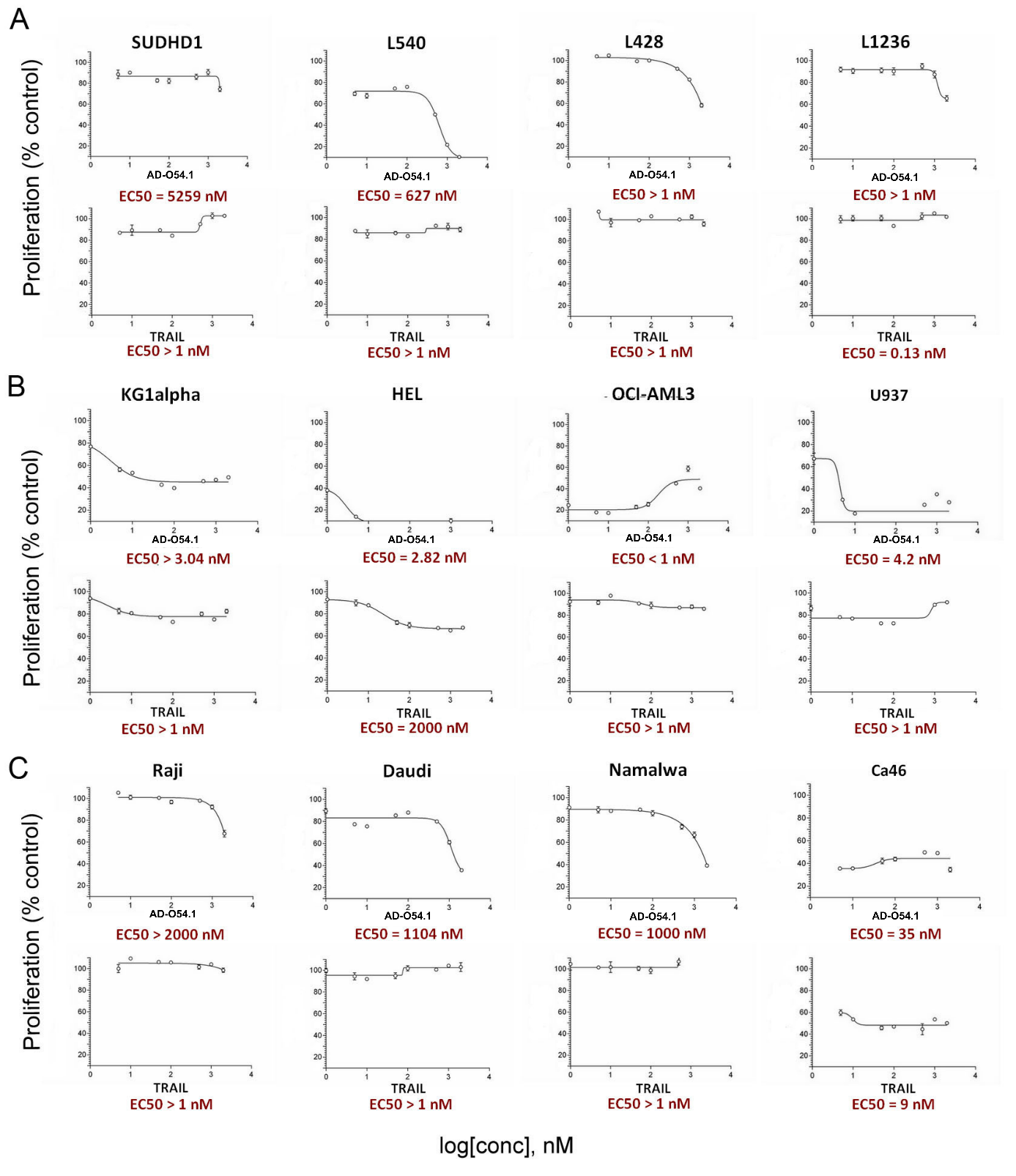


**Supplemental Figure 2**. AD-O51.4 and TRAIL dose-response curves and IC50 values in DLBCL cell lines.


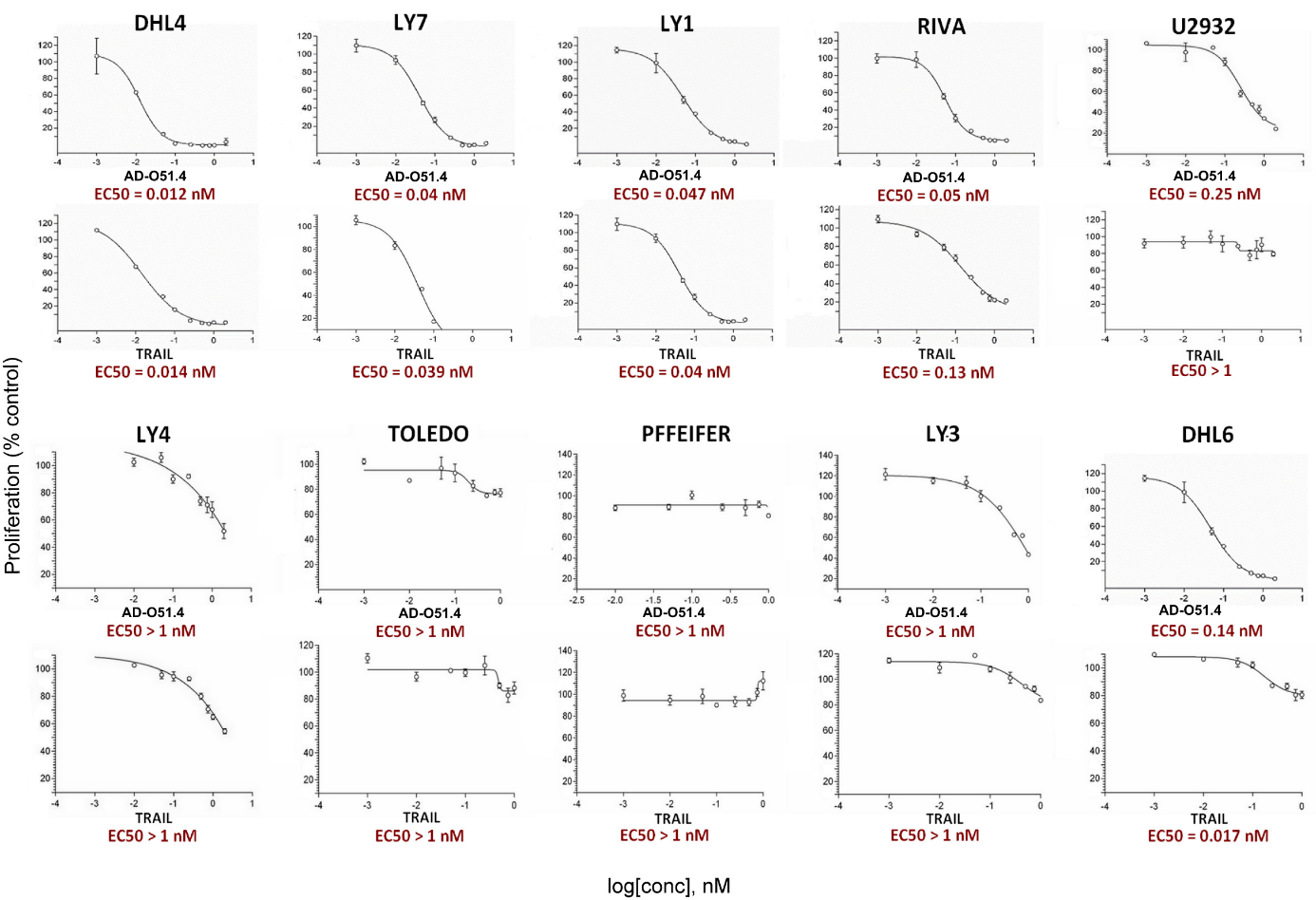


**Supplemental Figure 3.** Representative AnnexinV-PI staining scatterplots in sensitive and resistant lymphoma cell lines.


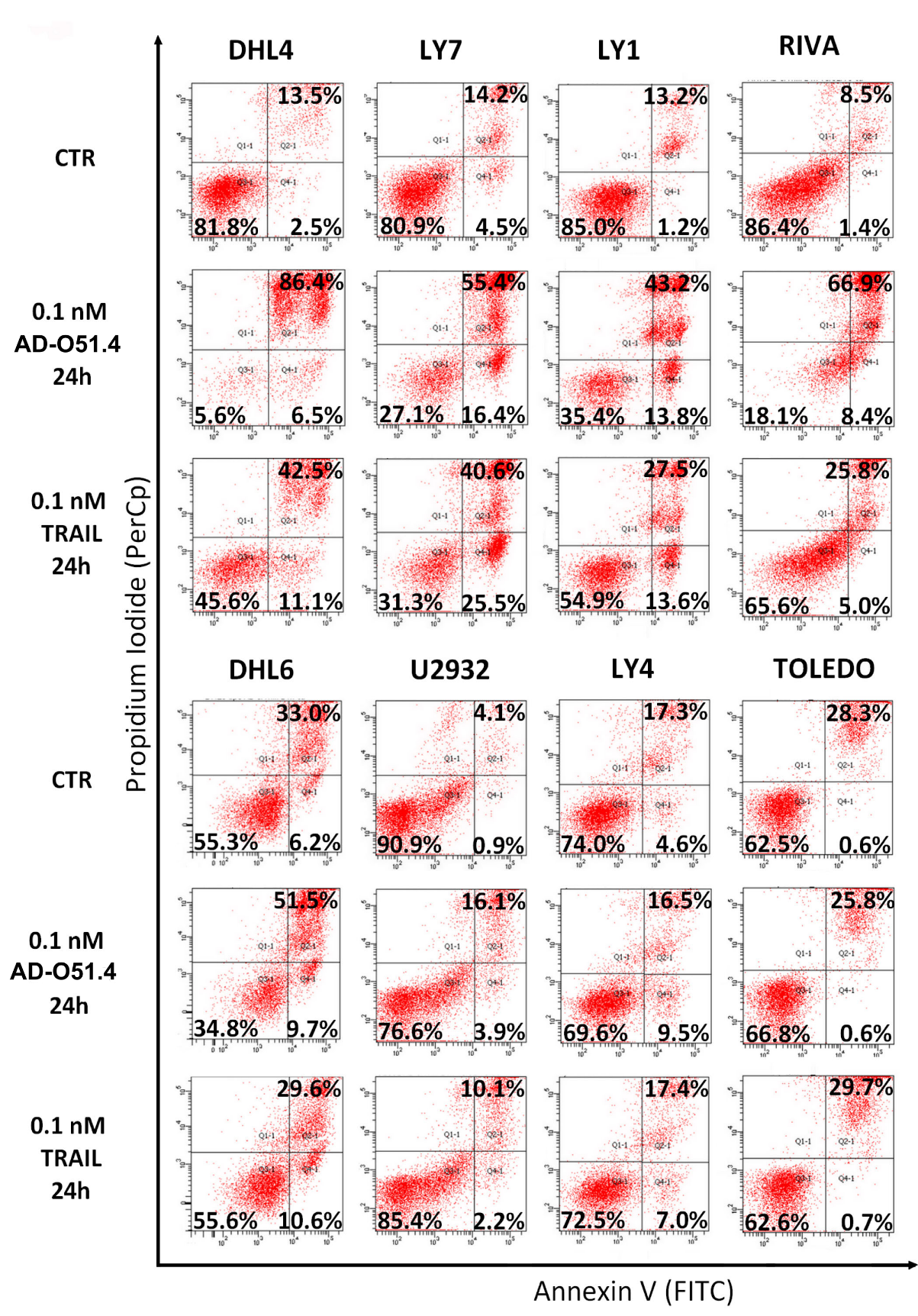


**Supplemental Figure 4**. AD-O51.4 and TRAIL sensitivity in GCB and ABC-type DLBCL lines. IC50 values are plotted on y axis, and cell lines are grouped according to GCB and ABC categories (x axis).For the cell lines with IC50 greater than 1, the IC50 values were set to 1. Cell line GCB and ABC designations were determined previously (18).


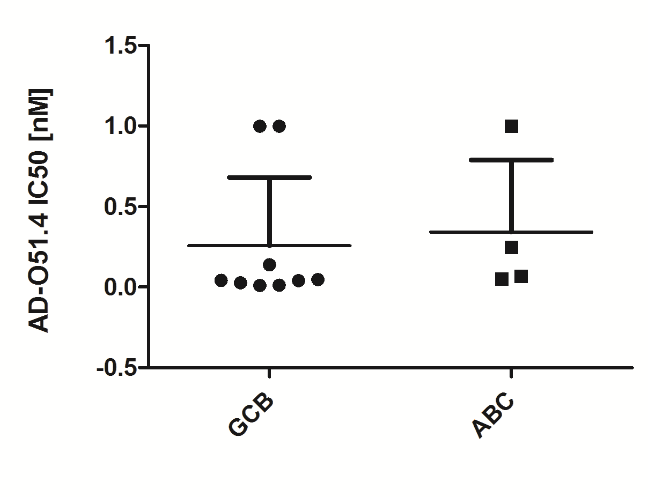

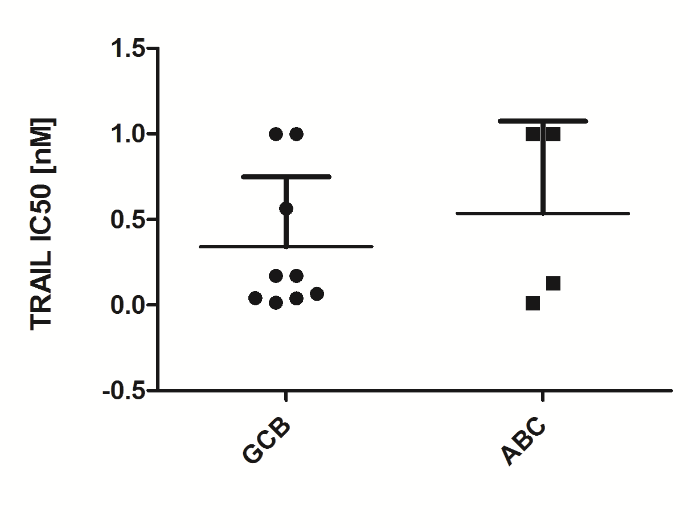


**Supplemental Figure 5**. Effect of membrane cholesterol elution on AD-O51.4 sensitivity in lymphoma cells. **A**. Cells were incubated (1h) with 10, 20 or 40 mM methyl-β-cyclodextrin (MβCD) to elute membrane cholesterol and disrupt lipid raft integrity. Depletion of cholesterol was confirmed with filipin staining (50 ng/mL) and flow cytometry. **B-C.** AD-O51.4 - sensitive LY1 and LY7 DLBCL cells were pretreated with MβCD and subsequently incubated with AD-O51.4. Apoptosis and viability were determined using AnnexinV/PI staining and MTS assay.


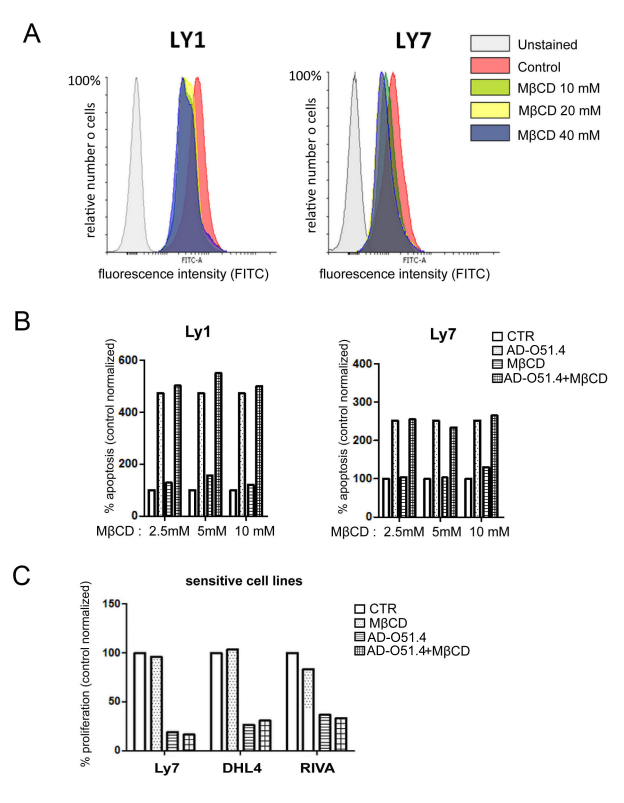

Supplement: Supplementary file 1 [file DataSheet_1.docx]
